# Mortality and Years of Life Lost in Diabetes Mellitus and Its Subcategories in China and Its Provinces, 2005–2020

**DOI:** 10.1155/2022/1609267

**Published:** 2022-04-22

**Authors:** Feixue Wang, Wei Wang, Peng Yin, Yunning Liu, Jiangmei Liu, Lijun Wang, Jinlei Qi, Jinlin You, Lin Lin, Maigeng Zhou

**Affiliations:** National Center for Chronic and Noncommunicable Disease Control and Prevention, Chinese Center for Disease Control and Prevention, Beijing, China

## Abstract

**Objectives:**

To analyze diabetes mellitus (DM) mortality and years of life lost (YLL) in different years and different subgroups at the national and regional levels in China from 2005 to 2020.

**Methods:**

We estimated mortality and YLL of DM and its subcategories for 31 provinces in China during 2005–2020 using multisource data from the National Mortality Surveillance System (NMSS).

**Results:**

The age standardized mortality rate (ASMR) of DM increased from 12.18 per 100,000 in 2005 to 13.62 per 100,000 in 2020, which was an increase of 11.86%. The ASMR of type 2 diabetes mellitus (T2DM) was much higher than that of type 1 diabetes mellitus (T1DM). The ASMR of T1DM remained stable, but the rate of T2DM increased, and the increase in male patients was higher than that in their female counterparts. At the same time, the burden of premature death was highest in the group ≥ 80 years old, and ASMR increased from 236.02 per 100,000 in 2005 to 358.86 per 100,000 in 2020. In 2005, the eastern region had the highest ASMR of DM, but the western region's ASMR grew faster and eventually became the highest in 2020. In addition, the YLL rate in the eastern region showed a downward trend; however, in the middle and western regions the YLL rate continued to rise, with that of the western region rapidly increasing.

**Conclusion:**

A dramatically upward trend in DM deaths can be seen in China from 2005 to 2020. DM remains a chronic disease in urgent need of prevention and control, especially in the elderly and people in less-affluent provinces. We must put forward more targeted policies to effectively allocate medical resources and focus on high-risk groups to reduce the premature-mortality burden of DM and its subcategories.

## 1. Introduction

Currently, noncommunicable diseases (NCDs) pose a threat to the health of the global population. In 2005, worldwide deaths from NCDs were 33 million; this number increased by 26% and reached 43 million in 2019 [1]. The World Health Organization (WHO) has identified diabetes mellitus (DM) as one of the four main NCDs meriting close attention [2]. The number of deaths globally due to DM increased by 48% during 2005–2019, from 2.0 million to 3.0 million. The International Diabetes Federation (IDF) estimates that there were 140 million people with DM in China in 2021 and that this number will rise to 174 million by 2045. Compared with high-income and low-income countries, the prevalence of DM increased significantly in middle-income countries [3].

In 2015, the United Nations General Assembly (UNGA) released its Sustainable Development Goals (SDGs), one of which was to reduce premature mortality from NCDs by one third by 2030 [4]. Due to geographical differences among provinces and problems in data acquisition, previous studies on the disease burden of DM in China have mostly focused on a certain groups of people in specific regions [5–8]. Therefore, studies that have been conducted to date on the national and regional burdens of premature mortality from DM in China are inadequate.

This necessitates an investigation into the burden of DM in China to supplement the existing relevant information and serve as a reference for public-health policymaking.

In this study, we estimated the cause-specific and premature-mortality rates of DM nationally and regionally during 2005–2020 using data from the National Mortality Surveillance System (NMSS) of the Chinese Center for Disease Control and Prevention (CDC). We identified trends and geographical variations in DM deaths in China, as well as sex differences and primary causes of change.

## 2. Materials and Methods

### 2.1. Data Sources

**Figure 1:**
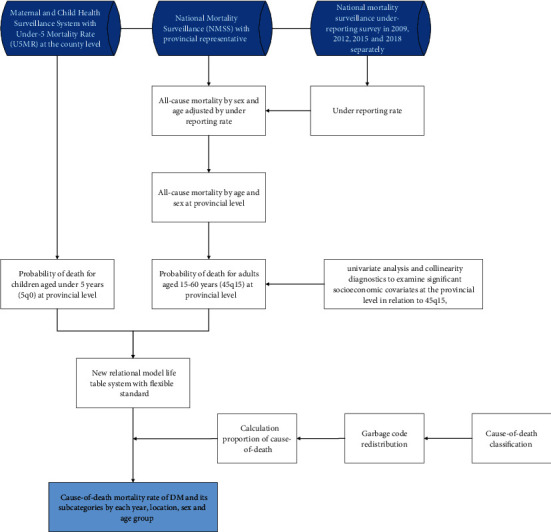


As stated above, data for all-cause mortality (ACM) and cause-specific mortality (CSM) was obtained from the NMSS. The NMSS monitors 605 surveillance points in 31 provincial-level administrative divisions that include more than 300 million individuals, accounting for 24% of China's population. It routinely collects detailed information on deaths in China in real time over the internet. The introduction of the NMSS has been described in detail elsewhere [9]. NMSS data were adjusted for underreporting in field surveys conducted for national mortality surveillance in 2009, 2012, 2015 and 2018 [10]. Those surveys collected underreporting data for the periods 2006–2008, 2009–2011, 2012–2014, and 2015–2017. To resolve substantial and systemic underreporting issues among children in lower-age groups, we used the Under-5 Mortality Rate (U5MR) at the county level, which was estimated from the 1982, 1990, 2000, and 2010 censuses; national surveys; Intra-Census Surveys; Maternal and Child Health Surveillance System; and the Disease Surveillance Point system (DSPs) [11]. All population surveillance and socioeconomic-covariate data were obtained from the National Bureau of Statistics. All rates were standardized by 2010 population census data. A flowchart of DM mortality estimates is shown in the Figure 1.

### 2.2. DM Mortality Estimation

#### 2.2.1. Estimation of All-Cause Mortality

By calculating the annual underreporting rate for each age/sex stratum among all surveillance points during 2006–2017, we used spline regression to predict this rate in each stratum in 2005 and 2018. ACM was adjusted by underreporting rate during 2005–2018. After fitting the time-varying trend of the results from 1996 to 2012, we predicted U5MR during 2013–2018 using a log-linear model. Quality control was then conducted by selecting a qualified range of ACM rates of 400–1000 per 100,000 persons as identifying outliers. Due to the discrete nature of each surveillance point, we used locally weighted regression by time and space. Weighting population counts in each surveillance point during 2005–2018, we calculated the age/sex ACM rate at the provincial level to generate the probability of death for children ≤ 5 years (5q0) of age and adults aged 15–60 years (45q15). We adopted univariate analysis and collinearity diagnostics to examine significant socioeconomic covariates at the provincial level in relation to 45q15, including urbanization rate (%), average years of educational attainment (unit), beds in medical institutions per 1000 persons, nonagricultural population (per 10,000 persons), and per capita gross regional product (GNP; yuan/person). We used a nonlinear mixed effect model to acquire a sex-specific 45q15 estimation at the provincial level during 2005–2018. An integrated suite of ASMRs (i.e., an abridged life table) for each location was produced by using a new relational-model life table system with a flexible standard based on the two parameters of 5q0 and 45q15 to generate a full set of age/sex-specific mortality rates for 31 provinces during 2005–2018 [12, 13].

#### 2.2.2. Estimation of Cause-Of-Death Mortality

*(1) Cause-Of-Death Classification*. Using the global burden of disease (GBD) method, we classified all causes of death into five levels. At Level 1, all disease burdens were split into three mutually exclusive categories: communicable, maternal, neonatal, and nutritional diseases (CMNNs); noncommunicable diseases (NCDs); and injuries. Level 2 classified Level 1 into 26 categories, including DM and kidney disease. DM was classified as Level 3. Level 4 described specific categories of DM, including T1DM and T2DM. The International Classification of Disease (ICD) codes of DM, T1DM, and T2DM are E10-E13.9, E10, and E11, respectively.

*(2) Garbage Code Redistribution*. “Garbage codes” refer to nonspecific codes for deaths (e.g., unspecified stroke), codes for deaths not caused by the disease of interest (e.g., cardiac arrest), or codes for deaths attributed to intermediate but not underlying causes of death (e.g., heart failure). We redistributed garbage codes by age, sex, location, and year to the most likely causes of death. This redistribution was ordered by regression models, based on fixed proportions, proportional reassignment, and fractional assignment of a death assigned to multiple causes, as discussed elsewhere by Naghavi et al. [14]

*(3) Calculated Proportion of Cause of Death*. By calculating the proportions of deaths reported by provinces, we expanded the coverage of death data included in our analysis. However, the coverage of death reporting in rural areas/nonhospitals in non-DSP areas was lower than in urban areas/hospitals. Therefore, we reweighted the proportions for each cause based on the proportion of hospitalized or nonhospitalized deaths and the urban or rural proportions in the DSP system by age, sex, and province [15].

*(4) Estimation of Cause-Of-Death Mortality*. We calculated the cause-of-death (CoD) mortality rate of DM and its subcategories by each year, location, sex, and age group by ACM rate and proportion of CoD calculated previously. To attenuate fluctuations in location-/year-/age-/sex-specific mortality where small numbers of deaths resulted in high variability of mortality between patterns across each stratum, we first conducted quality control (QC) by identifying outliers in each stratum, and then, we used spline regression to adapt trends of mortality rate over time and across space to fit cause-of-death (CoD) mortality rate within the same rubric.

#### 2.2.3. Aggregation and Central Rescaling of Causes of Death

Using a top-down hierarchical format consisting of five levels for ACM and the number of cause-specific deaths, we aggregated and rescaled causes of deaths for the period 2005–2018. For the first group, Level 1, the figure was the sum of CMNNs, NCDs, and injuries; i.e., the number of deaths from all causes. In each of the Level 1 categories, the estimated number of deaths at Level 1 represented total causes at Level 2. DM was geared to Level 3, which included T1DM and T2DM (Level 4). Because there was not enough information to assign Level 5 causes, the details of this study were limited to the Level 4 parent cause.

#### 2.2.4. DM Mortality Projection

Considering the stability of DM mortality and its subcategories according to previous results, we fitted a generalized linear model over time to forecast CSM in the same rubric in 2019 and 2020, by using the mortality results for DM and its subcategories in each location-/sex-/age-specific group stratum in 2015–2018. Finally, we calculated the change rate of the standardized mortality rate for DM during 2005-2020.

#### 2.2.5. Computation of YLL

Years of life lost is an index that shows premature mortality by applying a higher weight to deaths that occur in younger age groups, which are calculated as total deaths per age group multiplied by standard life expectancy for each age group. We selected the lowest observed risk of death for each 10-year age group in all populations > 5 million as the theoretical minimum-risk reference life table, which was equal to the standard life expectancy in YLL computation for DM and its subcategories during 2005–2020, and the theoretical minimum-risk reference life table was presented in Table 1 [16]. At the same time, we calculated the geographic variations of standardized mortality and the standardized YLL rate from 2005 to 2020.

## 3. Results

Table 2 shows estimated deaths, mortality rate, estimated YLLs, and YLL rate of DM and its subcategories by different subgroups in China in 2020 to reflect the total mortality rate of the population and to measure the risk of death from DM. Mortality and YLL of T2DM were about four times those of T1DM. Deaths from and YLLs of DM were higher in the eastern region than in other areas. The burden of DM increased with age, peaking in the age group ≥ 80 years.

**Figure 2:**
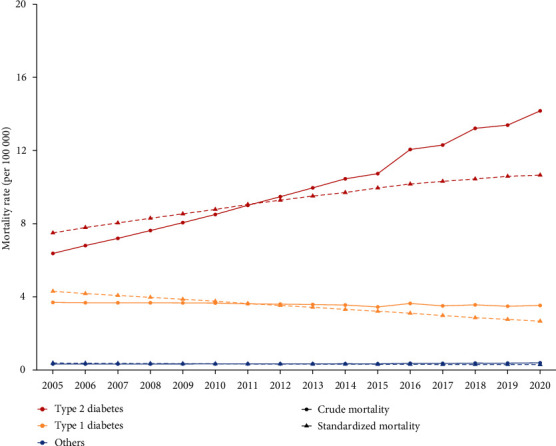
Trends in crude and age-standardized mortality rate of DM and its subcategories from 2005 to 2020.

**Figure 3:**
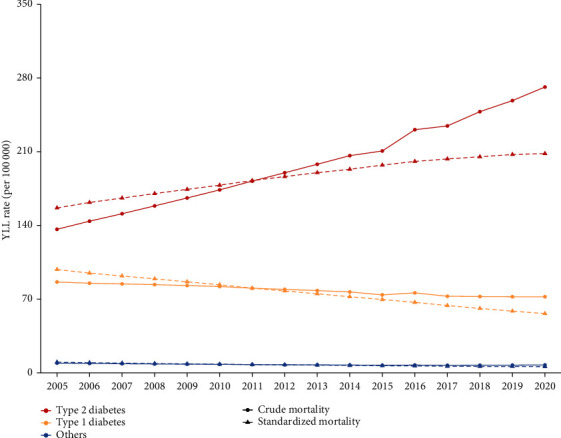
Trends in crude and age-standardized YLL rate of DM and its subcategories from 2005 to 2020.

Figures 2 and 3 demonstrate the time trends of crude and age-standardized mortality and YLL rate during 2005-2020. The ASMR and age-standardized YLL rate of T2DM increased faster than those of T1DM.

The ASMR and age-standardized YLL rates of DM in different subgroups and change rates between 2005 and 2020 are shown in Table 3, comparing the risk of death among different subgroups. The ASMR of DM increased by 12% from 12.18 per 100,000 in 2005 to 13.62 per 100,000 in 2020, and the age-standardized YLL rate increased by 2% from 264.79 person/years per 100,000 in 2005 to 270.38 person/year per 100,000 in 2020. In 2005, the ASMR of DM in the eastern region was highest, but the growth trend of ASMR in the western region (27%) over the past 16 years was much higher than that of the eastern region (1%). Therefore, by 2020, the western region replaced the eastern region as the one with the highest standardized mortality. Accordingly, the standardized YLL rate increased the most in the western region, by 14% from 2005 to 2020, while the eastern region showed a downward trend. The ASMR of males increased by 21%, much higher than that of females (3%), and the standardized YLL rate of men increased by 15% while that of women decreased by 11%. ASMR and standardized YLL rates showed a downward trend in people 0–69 years old, and the range of decrease increased along with age. However, the two age groups of 70–79 years and ≥80 years saw an upward trend, and the growth rate in the latter group was high as 52%.

**Figure 4:**
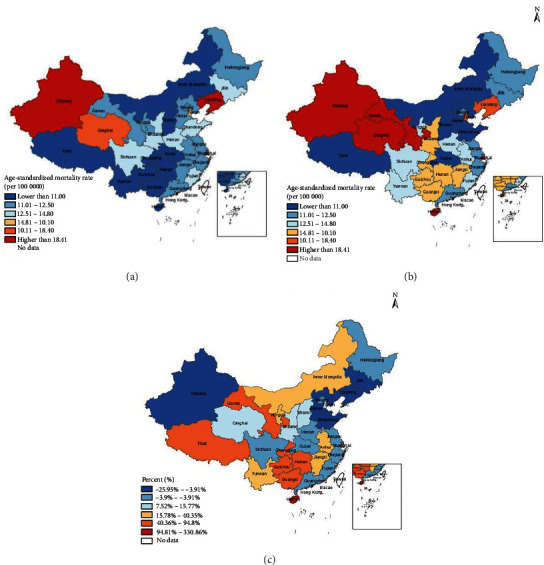
Provincial age-standardized mortality rate of DM and change rate in 2005 and 2020. (a) Provincial age-standardized mortality rate of DM in 2005; (b) provincial age-standardized mortality rate of DM in 2020; (c) change rate of provincial age-standardized mortality rate from 2005 to 2020.

**Figure 5:**
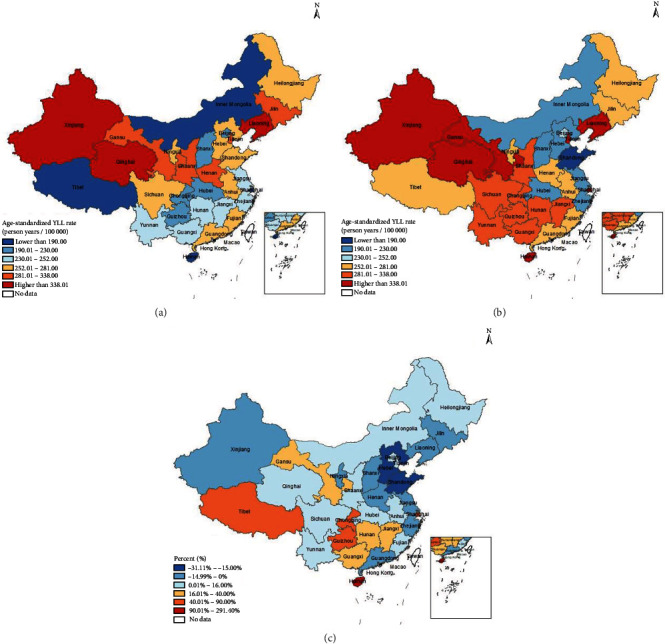
Provincial age-standardized YLL rate of DM and change rate in 2005 and 2020. (a) Provincial age-standardized YLL rate of DM in 2005; (b) provincial age-standardized YLL rate of DM in 2020; (c) change rate of provincial age-standardized YLL rate from 2005 to 2020.

Figures 4 and 5 demonstrate the provincial distribution and change rate of ASMR and the standardized YLL rate in 2005 and 2020. In the past 16 years, ASMR decreased in three provinces, the YLL rate decreased in 11 provinces, and other provinces showed upward trends. The provinces with the fastest rises in ASMR were Hainan, Chongqing, and Guizhou, and in YLL rate are Hainan, Shanghai, and Tibet. Meanwhile, Shandong, Liaoning, and Jilin had the great declines in ASMR and Shandong, Hebei, and Liaoning in YLL rate.

## 4. Discussion

Using the nationally representative NMSS data, we calculated the premature-mortality burden of DM in China, taking into account sex differences, age structure, and other drivers of its change. In general, DM mortality and YLL showed upward trends year by year, with older people accounting for nearly half of deaths. This demands attention as Chinese society begins to age.

We found that in 2020, the crude mortality and YLL rates of DM and its subcategories differed by a subgroup, which might have been due to reasons confirmed by the following research. A separate survey of 5838 Chinese adults in the 2009 China Health and Nutrition Survey showed that the Chinese visceral-adiposity index and body shape index were positively correlated with DM risk, indicating that obesity is a risk factor for DM [17]. Smoking, produced intake, and physical activity are also important factors affecting the risk of DM in the Chinese population [18–20]. Choice of lifestyle is affected by educational and economic levels, two factors that therefore cannot be ignored. Compared with the poor, the rich are at greater risk of DM, but socioeconomic factors also affect the likelihood of receiving treatment [21]. People with high levels of education have more knowledge about DM and are more likely to choose a healthy lifestyle. Moreover, diabetic patients in this category also have better drug compliance [22]. Research across different regions, age groups, and socioeconomic statuses in China has found that awareness of DM in the rural elderly is insufficient, which leads to a lack of treatment and control of the disease. The high prevalence and low awareness, control, and treatment rate of DM have increased the risk thereof in this population [23–27].

We also found that mortality of DM increased with age, peaking in the age group of ≥80 years in 2020. About 51% of patients diagnosed with DM are ≥65 years old [5, 28]. A 10-year cohort study showed significant excess mortality in older people with DM [29]. A study of the mortality trend in Hong Kong's diabetic patients in 2001–2016 showed that mortality in various age groups had decreased but was the highest in the ≥75-year age group. The difference between these results and those of the current study might be due to socioeconomic differences. However, the high mortality rate of the elderly diabetic population needs attention [30]. Advanced-age groups are prone to DM-related complications such as cardiovascular disease (CVD) and kidney disease. At the same time, these groups can have longstanding DM, in which mortality is higher than in newly diagnosed DM. In the prospective Atherosclerosis Risk in Communities (ARIC) study, researchers included 5791 older adults and compared mortality among patients with different durations of DM. The results showed that ACM rates were 21.2 per 1000 among those without DM, 33.8 per 1000 among those with recently diagnosed DM, and 48.6 per 1000 for those with longstanding DM. The CVD mortality rate was also higher for longstanding DM (17.3 per 1000) than for recently diagnosed DM (11.5 per 1000) [31]. Therefore, as age increases, so do the probabilities of DM complications and duration of disease, resulting in an increase in mortality.

After comparing ASMRs by sex in 2005 and 2020, we found that the ASMR of women in 2005 was higher, but the growth rate in men over the past 15 years was higher; thus, the trend was reversed by 2020. A study on the ACM rate in 2001–2016 based on the Hong Kong Diabetes Surveillance Database shows that in 2016, the ASMR of men was higher than that of women [30]. A study on the mortality trend of DM in China in 1990–2017 found that the mortality rate of males slightly exceeded that of females in the 2005–2017 interval, which is similar to the sex-based trend we studied [32]. The above results might be due to the higher awareness (50.6% in women, 42.5% in men, *P* < 0.001), treatment (42.6% in women and 35.5% in men, *P* < 0.001), and control (38.6% in women and 33.3% in men, *P* = 0.063) rates of DM in women, which lead to healthier lifestyles [26].

During 2005–2020, mortality and YLL rates showed upward trends, and T2DM increased more rapidly than T1DM. GBD results for 2019 demonstrated that the ASMR of DM in China trended downward from 2005 to 2019, and ASMR was 9.44 per 100,000 in 2019, which is lower than our results. This is because the ICD code of DM of GBD is different from our study, using E10-E10.1, E10.3-E11.1, E11.3-E11.9, and P70.2 [33]. Our code is E10-E13.9, which includes some complications that can be attributed to DM. This may be because the total number of deaths from these complications is annually increasing, leading to an increase in DM mortality. A forecast for DM in the US in 2030 also shows that from 2000 to 2010, mortality directly due to DM decreased by 40%, but the prevalence of DM increased, meaning that more people will have DM comorbidities in 2030 [34]. Therefore, the mortality of diabetic comorbidity might be on the rise.

We also found that the standardized mortality and YLL rates increased the most in the western region and the least in the eastern region from 2005 to 2020; the YLL rate decreased by 6% throughout China. This may be because the people in the eastern region are changing their living habits and improving their management of DM. However, the growth rate in the western region over the past 16 years has been great, meaning that attention should be paid to increasing health education and popularizing health knowledge in that region. The ASMR and YLL rates of people ages 0–69 years decreased during 2005–2020, but those of people ≥ 80 years old increased dramatically. Due to longer lifespans and longer duration of DM, the elderly were more likely to die of DM, which reflects the importance of prevention and control of DM in the elderly.

We found obvious geographical differences in the numbers of deaths caused by DM across different provinces. Although the ASMR and YLL rates of DM increased in most provinces from 2005 to 2020, the growth rate in Southwest China was higher, and the ASMR and YLL rates showed downward trends in Northeast China. This was closely related to living habits in different regions, which are related to the economic status of the corresponding province [35–37]. Due to the rapid economic development and high level of medical care in high-income areas such as Shanghai, the survival time of diabetic patients is longer, resulting in higher mortality in advanced-age groups. Conversely, in low-income areas such as Tibet, the survival time of patients with DM is reduced, so mortality is relatively lower.

In response to the rising trend of DM deaths, appropriate measures should be taken. Thanks to the development of the Healthy China 2030 strategy, public and medical institutions are focusing more on the prevention and control of DM. In clinical terms, we must improve the quality of medical care, increase the availability of drugs and enhance prognostic tracking and treatment of patients. Primary healthcare and public health can be complementary strategies in primary and secondary prevention by adjusting the focus of health services, making DM prevention and control a priority of basic health protection and designing effective population-based health interventions in the community. In the future, the entire DM prevention strategy should be considered in a broader sociopolitical and economic context to address the ingrained impact of the DM epidemic [38]. In addition, a healthy lifestyle can prevent DM to some extent, including but not limited to maintaining a healthy weight, exercising 30 min per day, quitting smoking, drinking moderately, and having a healthy diet [39–41].

Simultaneously, we should further strengthen the management and care of people with DM to control the disease's prevalence and mortality. In recent years, techniques widely used in DM management have included mobile phone applications and remote management, which have appeared to be more practical during the coronavirus disease 19 (COVID-19) pandemic [42]. The researchers of the present study have conducted in-depth studies on the characteristics and processes of different DM management methods, including remote monitoring of DM patients by professional medical staff [43–47]. In addition, health education should be strengthened to achieve the goals of self-management, self-protection, and self-care for diabetic patients [48–50].

This study had several limitations. First, the inferior quality of CoD data meant we might have underestimated the mortality of DM, but we tried to reduce this error through garbage code redistribution. Second, we did not break down the results by urban versus rural areas, because we roughly defined counties as rural areas and districts as urban areas. This vague classification might not have correctly explained urban/rural differences in guiding policies. Third, we did not specifically analyze the vital factor of deaths from various complications of DM; this study only focused on deaths directly caused by DM. Thus, we hope to research these complications further in the future.

## 5. Conclusion

In this study, we found that from 2005 to 2020, the premature mortality burden due to DM in China was on the rise, and the mortality rate of T2DM was higher and its upward trend was faster compared to T1DM. We found obvious sex and geographical differences in diabetes-related deaths. The burden was heavier in the elderly. An accurate estimation of DM mortality could provide empirical support for future DM prevention and control strategies, determine priorities in health protection, and provide a basis for accurate prevention and control of DM in different regions and groups.

## Figures and Tables

**Table 1:** Theoretical minimum risk reference life table.

Age	Life expectancy
0	87.885872
1	87.007248
5	83.035378
10	78.050774
15	73.069237
20	68.110138
25	63.157372
30	58.207291
35	53.27124
40	48.368408
45	43.49641
50	38.703121
55	33.98209
60	29.31563
65	24.73456
70	20.32095
75	16.09445
80	12.18093
85	8.7796783
90	6.0613198
95	3.8977709
100	2.2286451
105	1.6117361
110	1.363304

**Table 2:** The estimated deaths, mortality rate, estimated YLLs, and YLL rates of DM and its subcategories under different subgroups in 2020, China.

Subgroups	Estimated deaths				Mortality rate (/100,000)				Estimated YLLs (100,000 person years)				YLL rate (person year/100,000)			
	Total	Type 1	Type 2	Other	Total	Type 1	Type 2	Other	Total	Type 1	Type 2	Other	Total	Type 1	Type 2	Other
Total	253,968	49,580	198,882	5,506	18.09	3.53	14.17	0.39	4,929,541.12	1,014,619.92	3,809,806.72	105,114.48	351.15	72.27	271.38	7.49
Region																
East	107,910	14,592	91,991	1,326	18.44	2.49	15.72	0.23	2,002,567.22	293,789.76	1,683,897.03	24,880.42	342.23	50.21	287.77	4.25
Middle	75,237	21,668	51,492	2,077	17.22	4.96	11.79	0.48	1,512,916.27	449,211.47	1,025,731.25	37,973.55	346.30	102.82	234.78	8.69
West	70,821	13,320	55,398	2,102	18.55	3.49	14.51	0.55	1,414,057.63	271,618.69	1,100,178.44	42,260.51	370.36	71.14	288.15	11.07
Sex																
Male	123,487	23,990	96,929	2,568	17.22	3.34	13.52	0.36	2,570,719.18	531,735.55	1,985,972.97	53,010.66	358.45	74.14	276.91	7.39
Female	130,481	25,591	101,953	2,938	19.00	3.73	14.85	0.43	2,358,821.94	482,884.37	1,823,833.75	52,103.82	343.52	70.32	265.61	7.59
Age group (years)																
0-39	2,063	704	1,221	138	0.29	0.10	0.17	0.02	36,588.04	13,800.78	18,761.38	4,025.88	16.94	5.87	9.82	1.24
40-49	5,887	1,502	4,309	75	2.65	0.68	1.94	0.03	75,684.07	25,181.64	49,685.57	816.85	118.61	30.25	86.81	1.55
50-59	19,195	3,953	15,024	218	8.93	1.84	6.99	0.10	249,838.97	45,221.44	201,504.14	3,113.39	321.36	65.85	251.88	3.62
60-69	47,324	10,621	35,728	975	31.79	7.14	24.00	0.66	582,591.17	131,434.77	440,021.18	11,135.23	838.82	187.81	633.65	17.37
70-79	69,152	13,727	53,904	1,521	92.31	18.32	71.96	2.03	623,733.25	124,246.36	485,876.39	13,610.50	1,684.93	336.68	1,311.17	37.07
80+	110,347	19,073	88,696	2,578	358.86	62.03	288.44	8.39	790,386.44	142,999.39	627,985.09	19,401.97	4,371.20	755.55	3,513.51	102.14

**Table 3:** Age-standardized mortality and YLL rate of DM under different subgroups and change rate in 2005 and 2020.

Subgroups	Age-standardized mortality rate (/100,000)			Age-standardized YLL rate (person-year/100,000)		
	2005	2020	Change (%)	2005	2020	Change (%)
Total	12.18	13.62	11.86	264.79	270.38	2.11
Region						
East	12.83	13.02	1.47	269.71	252.45	-6.40
Middle	11.42	13.27	16.27	255.34	271.91	6.49
West	11.97	15.20	26.97	267.62	305.42	14.13
Sex						
Male	11.95	14.51	21.43	260.44	300.57	15.41
Female	12.38	12.73	2.83	268.94	239.89	-10.80
Age group (years)						
0-39	0.29	0.29	-0.47	18.51	16.94	-8.48
40-49	2.73	2.65	-2.93	122.14	118.61	-2.89
50-59	10.27	8.93	-13.07	368.77	321.36	-12.86
60-69	37.06	31.79	-14.22	989.85	838.82	-15.26
70-79	89.38	92.31	3.28	1,632.74	1,684.93	3.20
80+	236.02	358.86	52.04	2,874.95	4,371.20	52.04

## Data Availability

The data from national Disease Surveillance Point system in China are individual data, and some sensitive information needs to be kept confidential. Therefore, data sharing may not be realized.
